# Frequent detection of human polyomavirus 6 in keratoacanthomas

**DOI:** 10.1186/s13000-016-0509-z

**Published:** 2016-07-07

**Authors:** Jan Beckervordersandforth, Sreedhar Pujari, Dorit Rennspiess, Ernst Jan M. Speel, Véronique Winnepenninckx, Carlos Diaz, Wolfgang Weyers, Anke Maria Haugg, Anna Kordelia Kurz, Axel zur Hausen

**Affiliations:** Department of Pathology, GROW-School for Oncology & Developmental Biology, Maastricht University Medical Center, P. Debyelaan 25, P.O. Box 5800, 6202 AZ Maastricht, The Netherlands; Center for Dermatopathology, Freiburg, Germany; Medizinische Klinik IV, Universitätsklinikum Aachen, Aachen, Germany

**Keywords:** Human Polyomavirus 6, HPyV6, Keratoacanthoma, Non melanoma skin cancer, Fluorescence in situ hybridization, FISH

## Abstract

**Background:**

The recent discovery of the Merkel cell polyomavirus and its consistent association with Merkel cell carcinoma has drawn attention to the numerous recently discovered polyomaviruses and their possible involvement in the etiopathogenesis of non-melanoma skin cancer (NMSC). Data on the recently discovered human polyomavirus 6 (HPyV6) and its role in NMSC are sparse and in part controversial.

**Methods:**

In the present study we tested a large number (*n* = 299) of NMSC specimens for the presence of human polyomavirus 6 (HPyV6) by DNA PCR and HPyV6 fluorescence in situ hybridization (FISH). In detail, 59 keratoacanthomas (KA), 109 basal cell carcinomas (BCC), 86 squamous cell carcinomas (SCC) and 45 trichoblastomas (TB) were tested for the presence of HPyV6.

**Results:**

HPyV6 DNA PCR and subsequent sequence analysis revealed that 25 KAs (42.3 %), 23 BCCs (21.1 %), 8 SCCs (9.3 %) and 10 TBs (22.2 %) were HPyV6 positive. The presence of HPyV6 DNA was visualized and validated on the single cell level within the histomorphological context by HPyV6 fluorescence in situ hybridization.

**Conclusions:**

The high frequency of HPyV6 DNA in 42.3 % of KA possibly points to a role for HPyV6 in the etiopathogenesis of KAs. Although the detection rate of HPyV6 DNA in BCCs and TBs is within the previously reported detection range in normal skin, it does not exclude a possible role for HPyV6 in the carcinogenesis in a significant subset of these skin tumors.

## Background

Non melanoma skin cancer (NMSC) constitutes the most common group of human cancers and still its incidence is continuously rising [1, 2]. However, the underlying etiology and molecular pathogenesis of NMSC remains in large part unresolved. Immune senescence and immunosuppression have been identified as important risk factors in the pathogenesis of NMSC [3, 4], clearly pointing to a possible involvement of an infectious agent in NMSC etiology. In large epidemiological studies, an increased risk of cutaneous human papillomavirus (HPV) and cutaneous squamous cell carcinoma (SCC) was shown in the general population and immunosuppressed organ transplant recipients [5]. It was shown that the risk to develop squamous cell carcinoma (SCC), but not basal cell carcinoma (BCC) is associated with seropositivity for HPV [6]. Although the prevalence of the main HPV types found, i.e. β-HPV types 5 and 8 ranged between 27 and 85 % [7], they have been discussed as a possible co-factor in the early onset of cutaneous SCC, in combination with UV-induced DNA damage or immunosuppression [7]. Next to HPV, 13 human polyomaviruses (HPyV) are known (reviewed in [8, 9], of which 11 have been recently identified in neoplastic and non-neoplastic skin samples [10–14] and in other patient materials [9, 15–18]. Yet, no conclusive data for a role of the continuously growing number of human polyomaviruses in NMSC are available. Ever since their first detection, HPyV have repeatedly been incriminated with the etiopathogenesis of human cancers. However, only the recently discovered Merkel cell polyomavirus (MCPyV) has been identified as a new human tumor virus which is based on the consistent detection of integrated MCPyV DNA in the majority of Merkel cell carcinomas (MCC), a highly malignant NMSC [10–12]. In addition, tumor specific mutations within the large T antigen (LTag) of MCPyV are found in MCCs [13].

In 2010, human polyomavirus 6 (HPyV6) was isolated from skin swabs of healthy patients and characterized, but yet could not be linked to the pathogenesis of any human disease [14]. Although seroprevalence indicates that HPyV6 infection is common in adults, ranging from 69 to 76 % [14, 19, 20], it is detected in skin swabs of normal skins only between 14.3 and 27.6 % [14, 21]. Studies reporting the presence of HPyV6 DNA in NMSC are sparse [22–25], and in part controversial [21, 26, 27] (Table 1). Recently, a case of a keratoacanthoma (KA) which developed during treatment with Vemurafenib in a BRAF V600E positive melanoma patient was tested positive for the presence of HPyV6 [28] with pronounced viral load. In the present study we assessed the presence of HPyV6 DNA in a large number of NMSC specimens (*n* = 299), using HPyV6 DNA-PCR. In addition, we were able to visualize and validate the presence of HPyV6 DNA on the single cell level in a subset of HPyV6 DNA positive KAs, BCCs and SCCs by using fluorescence in situ hybridization (FISH).

**Table 1:** Summary of clinicopathological data and results of molecular investigation of non melanoma skin cancer

References	Tumor type	HPyV6 DNA	Detection Methode	HPyV6 IHC 6V32	HPyV6-FISH	Clinical Data
Schowalter et al. [14]	NS (*n* = 35)	14,3 %	DNA-PCR	NA	NA	IC
	Ser (*n* = 65)	69 %	VP1 ELISA			NA
Duncavage et al. [22]	MCC (*n* = 28)	3,5 %	rt-PCR	NA	NA	NA
Schrama et al. [24]	SCC (*n* = 21)	38 %	qPCR	NA	NA	NA
	BCC (*n* = 18)	5,5 %				
	MCC (*n* = 20)	10 %				
Scola et al. [25]	SCC (*n* = 52)	4 %	rt-PCR	NA	NA	IC
	BCC (*n* = 41)	7 %				
	KA (*n* = 42)	5 %				
Imajoh et al. [23]	NS (*n* = 34)	8,8 %	rt-PCR	NA	NA	NA
	SCC (*n* = 63)	3,2 %				
	BCC (*n* = 50)	2 %				
Nicol et al. [19]	Ser	37,5 % pos. (age 1–4)	VP1 ELISA	NA	NA	IC
		61,8 % pos. (age 15–19)				
		67,1 % pos (age 30–38)				
		98,2 % pos. (age 80+)				
Schrama et al. [28]	KA (*n* = 4)	25 %	rt-PCR	KA 1/4 (25 %)	NA	IS

*IS* immunosuppressed, *IC* immunocompetent, *PCR* polymerase chain reaction, *IHC* immunohistochemistry, *FISH* fluorescence in situ hybridisation, rt-PCR real time PCR, qPCR quantitative PCR, ELISA enzyme-linked immunosorbent assay, HPyV6 human polyomavirus 6, *NS* normal skin, *Ser* serum, *SCC* squamous cell carcinoma, *BCC* basal cell carcinoma, *KA* keratoacanthoma, *MCC* Merkel cell carcinoma, *NA* not applicable

## Methods

### Patients and tissues

Formalin-fixed and paraffin-embedded (FFPE) tissues of 299 skin excisions or biopsies were included in this study. All respective samples had been excised for diagnostic and/or therapeutic reasons. 51 BCC, 29 KA and 86 SCC were obtained from the Maastricht Pathology Tissue Collection (MPTC) and 58 BCC, 30 KA and 45 TB were obtained from the Center for Dermatopathology, Freiburg, Germany.

### DNA extraction

First, an H&E stain of the selected specimens was reviewed by four experienced pathologists (A.z.H., V.W. C.D., W.W.) to select paraffin material containing >95 % tumor tissue. Two consecutive 5 μm thick paraffin sections from each specimen were subjected to DNA extraction. In brief, after deparaffinization, the tissues were lysed by proteinase K overnight (56 °C) until complete tissue lysis, and DNA was extracted using the DNeasy Tissue kit (Qiagen). Purified DNA was measured in a spectrophotometer (Nano-drop, 2000, Thermo Scientific) and directly used for PCR. DNA quality and integrity was assessed by specimen control size (SCS) ladder as described [29].

### HPyV6 DNA-PCR

PCR was performed with 150 ng of genomic DNA using the AmpliTaq Gold (Roche) DNA polymerase in a final volume of 50 μl. For detection of HPyV6, primer sets and PCR conditions were used as described earlier [14]. Water instead of DNA template was used for PCR-negative controls containing all other PCR components.

### HPyV6 DNA sequence analyses

PCR products were submitted to automated nucleotide sequencing in an ABI 3130XL genetic analyzer (ABI). DNA sequences were compared and analyzed with the reference sequences of the National Center for Biotechnology Information (NCBI) Entrez Nucleotide Database gb gb|HM011563.1| (HPyV6 isolate 627a) and gb|HM011561.1| (HPyV6 isolate 607b) using the NCBI Blast program. Multiple sequence alignments were performed with Clustal omega (EMBL-EBI-2014).

### Detection of HPyV6 by fluorescence in situ hybridization (FISH)

FISH was performed as described earlier [30–32]. In brief, deparaffinized 3 μm thick tissue sections were pretreated with 0.2 M HCl, incubated with 1 M NaSCN and digested with 0.5 mg/ml pepsin (2500–3500 U/mg, Sigma Chemical, St. Louis, MO). The digoxigenin labelled specific whole genome HPyV6 DNA probe was generated by Nick translation from the pHPyV6-607 (gift from Christopher Buck Addgene plasmid # 24727) and added to the samples in a hybridization mixture, containing a concentration of 5 ng/μl, followed by denaturation of probe and tissue DNA (5 min, 80 °C) and hybridization overnight (37 °C, humid chamber, Thermobrite, Abbott, IL). Unbound HPyV6 DNA probe was stringently washed away. Bound probe was detected by sequential incubation of the following secondary antibody conjugates: Rhodamine-labeled sheep anti digoxigenine antibody (1:100; Roche, Basel, Switzerland) and Texas red-labeled donkey anti sheep secondary antibody (Brunschwig Chemie, Amsterdam, Netherlands). Prior to incubation, aspecific binding sites where blocked with Boehringer Blocking reagent (Roche). Cell nuclei were counterstained with 4.6-diamidino-2-phenylindole dihydrochloride (DAPI; 0.2 μg/ml, Vectashield, Vector Laboratories, CA). Signals were visualized using a DM 5000B fluorescence microscope (Leica, Wetzlar, Germany) coupled to an digital camera (Leica DC 300 Fx) for independent evaluation of FISH signals by 4 investigators (AzH, AMH, EJS, DR) according to criteria described earlier [31, 33].

## Results

### HPyV6-DNA PCR

**Fig. 1:**
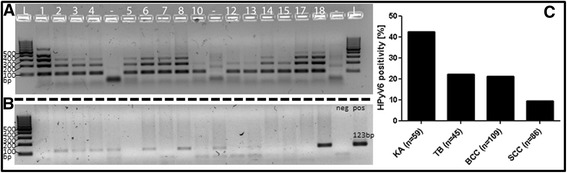
2 % agarose gel showing the specimen control size (SCS) ladder HPyV6 DNA-PCR and SCS ladder for keratoacanthoma (KA), results HPyV6 DNA-PCR: **a** reveals adequate DNA quality of KA in order to proceed with HPyV6 testing. **b** HPyV6 DNA PCR results of selected KA, showing amplification of the 123 bp fragment of the VP1 gene (123 bp) while using the primers according to Schowalter et al. [12] with the 123 pb positive controle. **c** Summary of the HPyV6-DNA PCR results on (KA), trichoblastoma (TB), basal cell carcinoma (BCC) and squamous cell carcinoma (SCC)

The DNA quality and integrity of extracted genomic DNA was assessed by specimen control size (SCS) ladder analysis (Fig. 1a) as described earlier [29]. All samples included in this study revealed sufficient DNA quality in order to test for HPyV6 by DNA PCR (Fig. 1a). HPyV6 DNA-PCR directed against the large T antigen (LTAg) of the HPyV6 genome revealed specific PCR products in 25/59 (42.3 %) of KA (Fig. 1b), 8/86 (9.3 %) of SCC, 23/109 (21.1 %) of BCC, and 10/45 (22.2 %) of TB. All PCR products were sequenced and confirmed the presence of HPyV6, revealing only minor nucleotide changes (<2 %).

### HPyV6-FISH

**Fig. 2:**
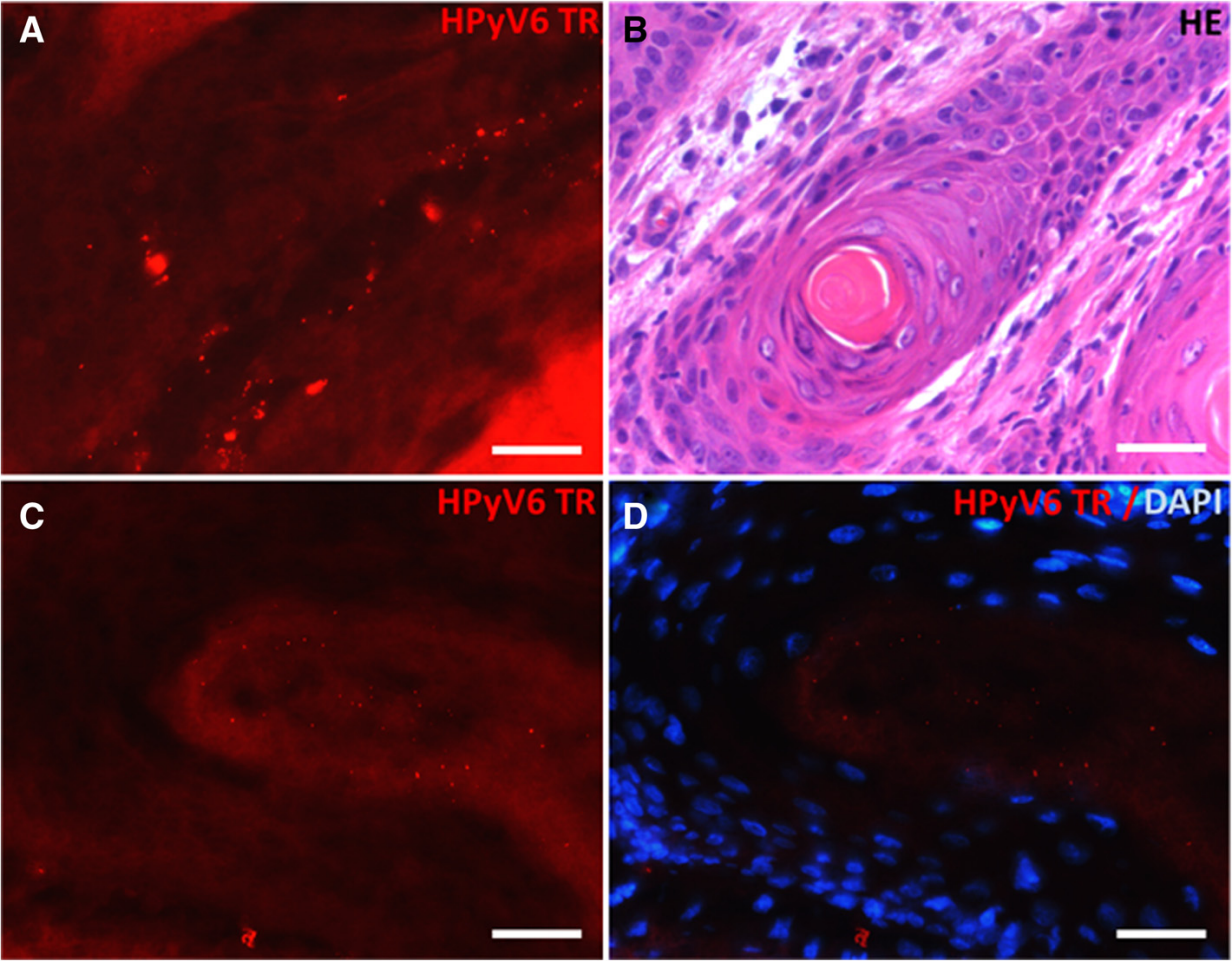
Photomicrographs of a representative example of the presence of HPyV6 detected by FISH in a keratoacanthoma: **a** DNA sequence nuclear hybridization signals in the keratinocytes of the lesion (red), located mainly in the middle and upper epidermis (scale bar 30 μm). **b** HE staining of keratoacanthoma used for HPyV6 FISH. **c** DNA sequence nuclear hybridization signals with dot-like specific positivity in the keratin layer of the lesion (red) (scale bar 30 μm). **d** overlay DAPI staining of nuclei of keratinocytes (blue) of the area of the lesion shown in C, showing no nuclei in the keratin layer with the positive FISH signals

In total, 26 KA were tested by HPyV6 FISH, including 13 HPyV6 DNA PCR positive cases and 13 negative cases. In none of the HPyV6 DNA negative KA specific HPyV6 fluorescence signals were found in the tumor cell nuclei. Out of 13 HPyV6 DNA positive KA, 8 (61.5 %) showed specific positive signals in the HPyV6 FISH. HPyV6 FISH hybridization signals were restricted to the mid- and upper epithelial layers of the KAs (Fig. 2a). In addition to these, specific dot-like signals in the keratin mass of the tumor (Fig. 2c) were seen. The positive FISH signals were restricted to the tumor areas, no specific signals were seen in the adjacent non-neoplastic epidermis. In dermal and subcutaneous tissue, specific HPyV6 FISH signals were found in perivascular and periadnexial lymphocytes (data not shown).

Pretreatment of the slides with DNAse lead to the disappearance of the specific HPyV6 FISH signals thus confirming the specificity of the hybridization signals. There was a highly significant correlation between HPyV6 DNA PCR and HPyV6 FISH results (*p* = 0.0007; Fisher’s exact test).

Out of 8 HPyV6 DNA PCR positive SCC, 2 were analyzed by HPyV6 FISH. In both cases, specific nuclear HPyV6 hybridization signals were seen within the tumor cells.

**Fig. 3:**
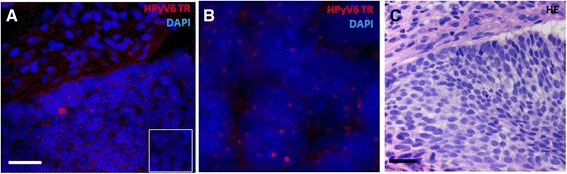
Photomicrographs of a representative example of HPyV6 detected by FISH in BCC: **a** Nuclear HPyV6 DNA hybridization signals in the epithelial tumor cells of a BCC (red), DAPI staining the nuclei (scale bar 30 μm). **b** Magnification of the marked quadrangular area in **a**. **c** HE staining of BCC used for HPyV6 FISH detection (scale bar 30 μm)

Also 5 of the HPyV6 DNA positive BCCs were subjected to HPyV6 FISH. In 4 out of the 5 HPyV6 DNA positive cases the presence of HPyV6 DNA was confirmed by HPyV6 FISH. HPyV6 FISH revealed the specific punctate nuclear hybridization pattern within the basaloid tumor cells (Fig. 3). This specific dot-like pattern was not seen in the adjacent non neoplastic tissues. Also here we could confirm the specificity of the used HPyV6 FISH probe by DNAse pretreatment.

## Discussion

Although major contributions to the understanding of the pathogenesis of NMSC have been made in the past decades, the etiology of most NMSC remains elusive. The discovery of the Merkel cell polyomavirus in 2008 and its role in the etiopathogenesis of Merkel cell carcinoma has drawn the attention to the continuously growing number of newly characterized human polyomaviruses [10–13] (reviewed in [8]).

In the present study we aimed to comprehensively assess the presence of HPyV6 DNA in a large cohort of NMSC by testing tumor specimens by HPyV6 DNA PCR. Previous studies, mainly using rtPCR/qPCR [19, 21–27] (Table 1) in smaller NMSC cohorts revealed variable positivity for HPyV6 in diverse types of NMSC with varying viral copy numbers, suggesting no evident pathological role of HPyV6 in NMSC. It is of interest that by using HPyV6 DNA PCR in this study we were able to detect more HPyV6 DNA positive NMSC as previously reported (Table 1). In addition, we established an HPyV6-specific FISH on formalin fixed and paraffin embedded material. The HPyV6 FISH enabled us to visualize the HPyV6 DNA on the single cell level within the histomorphological context of the diverse types of NMSCs.

Yet, the HPyV6 status of KA had solely been investigated in one larger study of 42 cases, showing only 2 positive cases (5 %) [25]. The underlying explanations for the obviously discrepant results with the present of HPyV6 prevalence in KA is difficult to understand. It seems unlikely that the difference is due to technical reasons since in the other study qPCR was used, which has been shown to provide a comparable sensitivity as the DNA PCR used in the present study. Most likely the selection of the targeted sequence of the HPyV6 genome impacts the detection rate of HPyV6 in these skin lesions. In this study we used primers targeting the LTAg region of HPyV6, according to Schowalter et al. [14]. Duncavage et al. [22] used three different qPCR targeting two times different regions of the LT-AG and one time the VP1 region, Schrama et al. [24] used qPCR targeting the LTAg, Imajoh et al. [23] used qPCR targeting the LTAg and the VP3 region and Schrama et al. [28] used pPCR targeting the VP3 region.

Of interest, Schrama et al. [28] investigated the presence of HPyV6 in Vemurafenib induced epithelial proliferations. Vemurafenib is a BRAF-specific inhibitor used in the therapy of BRAF mutated melanoma patients (reviewed in [34]). The authors reported a HPyV6 positive KA, which developed under Vemurafenib therapy in a BRAF V600E positive melanoma patient, with a pronounced viral load. Since multiple studies have reported that KA and SCC are one of the most frequent severe adverse side effects due to Vemurafenib this is of particular interest (reviewed in [35]). It has recently been described that HPV can cooperate with Vemurafenib to promote the initiation of some cutaneous tumors [36], which basically might be postulated for HPyV6 in KAs as well. Furthermore, KA frequently occur and relapse under immunosuppression [4], which relates the occurrence of these tumor to an impaired immune system. The high prevalence of HPyV6 in KA in our study may point to HPyV6 playing a pathogenic role in the development of cutaneous malignancies in the context of immune suppression or immune senescence. This is indirectly supported by the rather infrequent finding of HPyV6 in other NMSC.

The HPyV6-specific FISH hybridization pattern in KA, i.e. its presence in mid- and upper epithelial part and within the keratine layer, resembles a pattern which has previously been described for some human papilloma viruses (HPV) in skin tumors [37]. It has been shown that HPV DNA is commonly found in superficial layers of skin tumor lesions, but is not necessarily present throughout the whole tumor [38]. This may contribute to a low viral load in the proliferative active cells. During maturation of these cells the viral load increases and exceeds the detection limit, becoming detectable in the superficial layers of the tumor lesion.

Also the HPyV6 status in SCC has recently been investigated in 3 different studies, reporting a broad variation in positivity, ranging between 3.2 % [23] and 38 % [24]. In all the three studies rtPCR/qPCR was used to detect HPyV6 DNA. In the present study, 9.3 % of tested SCC were positive for HPyV6. These values are closer to the data of Scola et al. [25] and Imajo et al. [23] who found HPyV6 DNA in 3.2 and 4 % of the SCC samples. In comparison with the other tumors we investigated, the prevalence of HPyV6 in SCC is rather low, lower [14] or comparable [23] to the prevalence of HPyV6 in normal skin, suggesting no pathological role in the development of SCC in immune competent patients.

The prevalence of HPyV6 in BCC is of interest because three different studies reported HPyV6 positivity ranging between 2 % [23] and 7 % [25]. In all these studies the presence of HPyV6 was assessed by qPCR, whereas we used the HPyV6 DNA PCR according to Schowalter et al. [14]. Beside BCC, we also tested HPyV6 in TB which yet had not been done before. TB are benign neoplasms of follicular differentiation, which share several histomorphological features with nodular BCC, thus hampering the ease of histomorphological diagnostics in certain circumstances [39]. In our study 21.1 % of BCC and 22.2 % of TB were shown to harbor HPyV6 DNA. A study [14] using HPyV6 DNA PCR, showed that skin swabs of healthy donors were in 14.3 % positive for HPyV6 DNA. This may suggest that the choice of the target sequence used in qPCR might contribute to the different results in comparison to our findings, generated by using conventional DNA PCR. To validate these positive results we combined the HPyV6 DNA PCR with HPyV6 FISH also in BCC and found HPyV6 specific hybridization signals within the BCC tumor cells (Fig. 3).

## Conclusions

We demonstrate the presence of HPyV6 DNA in a large cohort of NMSC by PCR and HPyV6 FISH. The introduction of an HPyV6-specific FISH on FFPE tissues is a powerful tool to analyze the presence of HPyV6 DNA on the single cell level within the histomorphological context. We identified HPyV6 frequently in KA, and in a significant subset of BCC and TB and to a far lesser extent in SCC. The significant association with KA is remarkably, as the virus reveals a distribution pattern that has been described for pathogenic HPV infection in skin tumors. The high frequency of HPyV6-DNA in 42.3 % of KA might point to a role for HPyV6 in the etiopathogenesis of KAs. It would be highly interesting to test a larger number of Vemurafenib induced KAs and SCCs for the presence of HPyV6. In addition, HPyV6 might play a role in the carcinogenesis of a significant subset of BCC and TB.

## Abbreviations

BCC, basal cell carcinoma; FISH, fluorescence in situ hybridization; HE, hematoxylin eosin; HPV, human papillomavirus; HPyV6, human polyomavirus 6; HPyV7, human polyomavirus 7; KA, keratoacanthoma; LT-Ag, large T-antigen; MCC, Merkel cell carcinoma; MCPyV, Merkel cell polyomavirus; NMSC, non melanocytic skin cancer; SCC, squamous cell carcinoma; SCS, specimen control size; TB, trichoblastoma; VP1, Viral protein 1; VP3, Viral protein 3.
